# Altered immune cell in human severe acute pancreatitis revealed by single-cell RNA sequencing

**DOI:** 10.3389/fimmu.2024.1354926

**Published:** 2024-09-20

**Authors:** Zheyi Wu, Shijie Wang, Zhiheng Wu, Junjie Tao, Lei Li, Chuanming Zheng, Zhipeng Xu, Zhaohui Du, Chengpu Zhao, Pengzhen Liang, Aman Xu, Zhenjie Wang

**Affiliations:** ^1^ Department of Emergency Surgery, The First Affiliated Hospital of Bengbu Medical College, Bengbu, China; ^2^ Department of General Surgery, Huangshan City People’s Hospital, Huangshan, China; ^3^ Department of Gastrointestinal Surgery, The First Affiliated Hospital of Anhui Medical University, Hefei, China; ^4^ Institute of Acute and Critical Care, The First Affiliated Hospital of Bengbu Medical College, Bengbu, China

**Keywords:** severe acute pancreatitis, single cell RNA-sequencing, inflammatory, monocytes, transcriptomic analyses

## Abstract

**Background:**

Severe acute pancreatitis (SAP) is characterized by inflammation, with inflammatory immune cells playing a pivotal role in disease progression. This study aims to understand variations in specific immune cell subtypes in SAP, uncover their mechanisms of action, and identify potential biological markers for predicting Acute Pancreatitis (AP) severity.

**Methods:**

We collected peripheral blood from 7 untreated SAP patients and employed single-cell RNA sequencing for the first time to construct a transcriptome atlas of peripheral blood mononuclear cells (PBMCs) in SAP. Integrating SAP transcriptomic data with 6 healthy controls from the GEO database facilitated the analysis of immune cell roles in SAP. We obtained comprehensive transcriptomic datasets from AP samples in the GEO database and identified potential biomarkers associated with AP severity using the “Scissor” tool in single-cell transcriptomic data.

**Results:**

This study presents the inaugural construction of a peripheral blood single-cell atlas for SAP patients, identifying 20 cell subtypes. Notably, there was a significant decrease in effector T cell subsets and a noteworthy increase in monocytes compared to healthy controls. Moreover, we identified a novel monocyte subpopulation expressing high levels of *PPBP* and *PF4* which was significantly elevated in SAP. The proportion of monocyte subpopulations with high *CCL3* expression was also markedly increased compared to healthy controls, as verified by flow cytometry. Additionally, cell communication analysis revealed insights into immune and inflammation-related signaling pathways in SAP patient monocytes. Finally, our findings suggest that the subpopulation with high *CCL3* expression, along with upregulated pro-inflammatory genes such as *S100A12*, *IL1B*, and *CCL3*, holds promise as biomarkers for predicting AP severity.

**Conclusion:**

This study reveals monocytes’ crucial role in SAP initiation and progression, characterized by distinct pro-inflammatory features intricately linked to AP severity. A monocyte subpopulation with elevated *PPBP* and *CCL3* levels emerges as a potential biomarker and therapeutic target.

## 1 Introduction

Acute pancreatitis (AP) is characterized by acute inflammation and damage or necrosis of pancreatic tissue due to the activation of pancreatic enzymes within the pancreas (1). Diagnosis is typically established through the observation of a serum amylase level elevated to at least three times the upper limit of the normal range, coupled with persistent epigastric pain (2). Severe acute pancreatitis (SAP) represents one of the most critical forms of AP (3), SAP is primarily characterized by the persistence of organ failure for over 48 h. At present, the primary approach for treating SAP remains supportive therapy. The absence of distinctive early diagnostic indicators and specific targeted therapeutic interventions has led to a high mortality rate associated with SAP (4, 5).

In investigations concerning the pathogenesis of pancreatitis, it has been determined that pancreatic injury is a multifactorial process (6). Inflammatory responses and dysregulated immune processes subsequent to tissue damage are considered among the foremost factors contributing to the development of SAP (7, 8). As early as 1987, it was proposed that pancreatic enzymes might serve as contributing factors to SAP rather than being its primary cause, with the leading culprit being the over-activation of leukocytes (9). Relevant studies have shown that immune cells initiate infiltration within the first few minutes of SAP. Early damaged pancreatic acinar cells collaborate in inducing systemic inflammatory response syndrome and other disease complications by releasing cytokines, chemokines, and immune cells (10, 11). Subsequent research further supports the view that immune system dysregulation is the primary cause of systemic complications in AP (12, 13). It also plays a pivotal role in determining the severity and prognosis of the condition (12, 14). Furthermore, two recent studies have highlighted that a prominent characteristic of SAP is a significant dysregulation of the immune system (7, 15). In this study, we employed RNA sequencing (RNA-seq) technology to identify substantial differences in the expression of immune factors that impact the severity of AP. Increased expression of genes such as *S100A8*, *S100A9*, and *MMP25* proved to be highly effective in predicting the onset of SAP (15).

While the application of this technique has illustrated the potential mechanisms through which circulating immune cells promote SAP, there are limitations in comprehensively investigating changes in immune cell composition and its molecular profile in SAP patients. In contrast to traditional RNA-seq methods, single-cell RNA sequencing (scRNA-seq) analyzes the transcriptional information of individual cells rather than cell populations, enabling a more precise delineation of intercellular heterogeneity (16). It can be harnessed to investigate a wide array of biological information, encompassing the transcriptome, genome and epigenetics (17, 18). Hence, to gain deeper insights into the functions of diverse immune cell subpopulations and their molecular alterations in SAP, this study represents the first attempt to employ scRNA-seq in the context of SAP. It offers a comprehensive exploration of the biological features of circulating immune cells at the molecular level, with the aim of providing a more intuitive and innovative approach for the precise prediction of SAP progression and targeted therapeutic interventions.

## 2 Materials and methods

### 2.1 Study subjects

A total of 9 untreated SAP patients and 2 healthy donors were enrolled in this study, and those patients with SAP were enrolled within 24 h. All study participants were recruited from the First Affiliated Hospital of Bengbu Medical College. The scRNA-seq analysis was conducted using data from 7 SAP patients (male/female = 2/5, mean age 58 years), with control data obtained from 7 healthy donors from GSE190510 and GSE175499 in The Gene Expression Omnibus database(GEO), However, during the subsequent data quality control and cell filtering stages, we found that the number of cells in one of the preprocessed datasets differed significantly from those in the other datasets. To avoid bias due to differences in the quality of the control group data, we excluded this dataset from further analysis. Therefore, in our subsequent analyses, we used data from the remaining 6 healthy controls across the other two datasets. (male/female = 2/4, mean age 62 years). Flow Cytometry Staining was performed using data from 2 SAP patients (male/female = 1/1, mean age 47 years) and 2 healthy donors (male/female = 1/1, mean age 47 years), The characteristics of the enrolled patients with SAP patients and control data are shown in **Supplementary Tables S1**, **S2**. The diagnosis of SAP primarily relies on the revised classification criteria established in accordance with the 2021 Chinese Guidelines for the Diagnosis and Treatment of Acute Pancreatitis and the Atlanta Classification Standards (19). All SAP patients and healthy donors included in the study provided written informed consent, and the study received ethical approval from the Ethics Committee of the First Affiliated Hospital of Bengbu Medical College (Ethical Approval Number: 2022KY014).

### 2.2 Blood collection, scRNA-seq and flow cytometry staining

#### 2.2.1 Blood collection

A total of 2.5 ml of fresh peripheral blood was separately collected from SAP patients and healthy controls. The blood samples were processed within 2 h. Peripheral blood mononuclear cells (PBMCs) were extracted using a single-cell separation solution (Tianjin Haoyang), retaining cells that were stained with Trypan Blue and had a viability greater than 90%. These cells were then used for subsequent experiments. Following the manufacturer’s instructions, the cells were converted into a suspension using a cell separation solution kit(8804-6837-74; Thermo Fisher Scientific), allowing for the thorough enrichment of single-nucleus cells for scRNA-seq and flow cytometry experiments.

#### 2.2.2 ScRNA-seq

The quality-checked PBMCs were processed into single-cell suspensions and loaded onto the Chromium Controller (10x Genomics, Pleasanton) to generate Gel Bead-in-Emulsion (GEMs). Barcoded sequencing libraries were prepared following the instructions of the Chromium Single Cell 3’ Reagent Kit v3 (10x Genomics, Pleasanton). After library preparation was completed, sequencing was performed on the Illumina NovaSeq 6000 platform.

#### 2.2.3 Flow cytometry staining

Collect fresh peripheral blood from SAP patients and healthy controls using appropriate anticoagulant tubes. Take 100 μl of anticoagulated blood and mix it in a flow tube. Add 5 μl of Anti-Human CD14, PE (Clone: 61D3) flow antibody (F1101402, MULTISCIENCES, Hangzhou, Zhejiang, China), 2 μl of CCL3 antibody (1:50, ab307620, Abcam, Cambridge, UK), and 5 μl of CCL5 antibody (1:20, 515506, BioLegend, San Diego, CA, USA). Mix thoroughly and incubate in the dark at room temperature for 20 min. Then add 500 μl of 1× red blood cell lysis buffer (LSC, MULTISCIENCES, Hangzhou, Zhejiang, China), vortex, and mix well, and incubate in the dark at room temperature for 20 min. Add 2 ml of PBS, centrifuge at 400 ×g for 5 min. Discard the supernatant, add 0.5 ml of flow staining buffer, resuspend, and analyze on a flow cytometer. Flow cytometry data analysis was performed using FlowJo v10.

### 2.3 ScRNA-seq data analysis

We processed the SAP patient data matrix and the data matrix from healthy individuals in GEO using “Cellranger” and integrated them. Further data processing was performed using the “Scanpy” package (Version 1.9.3) (20). Initially, data filtering was applied, retaining cells with a gene count greater than 500 and less than 6000, with mitochondrial genes accounting for less than 10% of the total genes, while removing cells that did not meet these criteria. We then used the “Doublet Detection” package (Version 2.4) to eliminate doublets. If the expected doublet rate was set at 0.1, cells with a doublet score greater than 0.5 were removed (21). In addition, This study followed the approach of Zhang Zemin’s team, which involves filtering out all ribosomal, mitochondrial, and immunoglobulin genes (22). By setting a blacklist of genes, we removed non-inflammatory genes that remained in the expression matrix after preprocessing and could affect cell clustering, such as immunoglobulin genes, T-cell receptor genes, and ribosomal genes (as shown in **Supplementary Table S3**). In the dimensionality reduction analysis, Principal Component Analysis (PCA) was performed on the top 2500 highly variable genes calculated using the “Scanpy” package. A k-nearest neighbors (k-NN) graph was constructed in the space of 30 principal components using the Euclidean distance (k = 10). Unsupervised clustering of cells was carried out using the Leiden method. The Harmony package was then utilized to remove batch effects between sample (23). After batch effect removal, at a resolution of 0.5, the unsupervised clustering results were visualized as distinct cell clusters using the Uniform Manifold Approximation and Projection (UMAP) algorithm. Different cell subpopulations were defined based on the previously reported marker genes of specific cell types. Each subpopulation was named based on the specific marker genes identified for that subpopulation.

### 2.4 Differential expression genes analyses

We used the “Scanpy” package and Wilcoxon rank sum test to conduct differential analysis of cell subsets. We considered values with log-fold change (logfc) greater than 2 and p-value less than 0.05 as significant, resulting in the identification of differential genes between SAP and healthy control datasets in cell subsets. For gene ontology (GO) and Kyoto Encyclopedia of Genes and Genomes (KEGG) pathway enrichment analyses, we employed the “ClusterProfiler” package (Version 4.6.0). Differential analysis of cell subpopulations based on the Wilcoxon rank-sum test was performed using the “Scanpy” package, with a threshold of logfc greater than 2 and a p-value less than 0.05. This analysis helped identify differential genes between SAP and healthy control datasets within the cell subpopulations. To perform GO and KEGG pathway enrichment analysis, we utilized the “ClusterProfiler” package (Version 4.6.0) in R.

### 2.5 Cell–cell communication analysis

We employed the “CellChat” package (Version 1.6.0) in R for cell-to-cell inference and analysis. This package is designed to predict the major signal inputs and outputs for cells and understand how cells and their signals coordinate their functions. It does so through network analysis and pattern recognition methods, with a primary focus on analyzing scRNA-seq data (24). To begin, the “CellChat” package was separately applied to the scRNA-seq datasets of the SAP group and the control group. It calculated the relative expression levels of receptor and ligand genes within each cell in the dataset. This analysis identified specific cell subpopulations, signaling pathways, and ligand-receptor pairs in which biological functions were altered, and the results were visualized.

### 2.6 Scissor selected cells

By integrating the PBMC whole transcriptome data from GSE194331, which includes AP patients with different severity classifications, and the PBMC single-cell transcriptome data from the 7 SAP patients obtained in this study, we established a phenotype-optimized correlation matrix regression model using the “Scissor” (Single-cell Identification of Subpopulations with Bulk Sample Phenotype Correlation) package (25). This model was constructed based on the phenotype-related data available in the extensive dataset from GSE194331.The primary objective of this model was to identify the most relevant subpopulations associated with SAP within the single-cell sequencing samples. It further aimed to investigate the mechanisms influencing the progression of acute pancreatitis and their impact on disease severity classification. Cell types that exhibited a positive correlation with the phenotype were labeled as Scissor^+^ cells, while those with a negative correlation were labeled as Scissor^_^ cells. Subsequently, in the downstream analysis, we further characterized the Scissor^+^ cells.

### 2.7 Statistical analyses

The analysis was conducted using R (Version 3.6.1; http://www.R-project.org, R Foundation for Statistical Computing, Vienna, Austria) and Python software. Categorical variables were expressed as counts and proportions (%). The normality of data was assessed using the Kolmogorov-Smirnov test. Continuous variables were presented as mean ± SD. Comparisons between groups of continuous variables were performed using Student’s t-test or Mann-Whitney U test, while categorical results were analyzed using the chi-squared test or Fisher’s exact test. The significance level was set at a two-tailed p-value of 0.05. DEGs between conditions in this study were calculated with the Wilcoxon rank-sum test (two-tailed).

## 3 Results

### 3.1 ScRNA-seq analyses revealed changes between patients of SAP and healthy control

**Figure 1:**
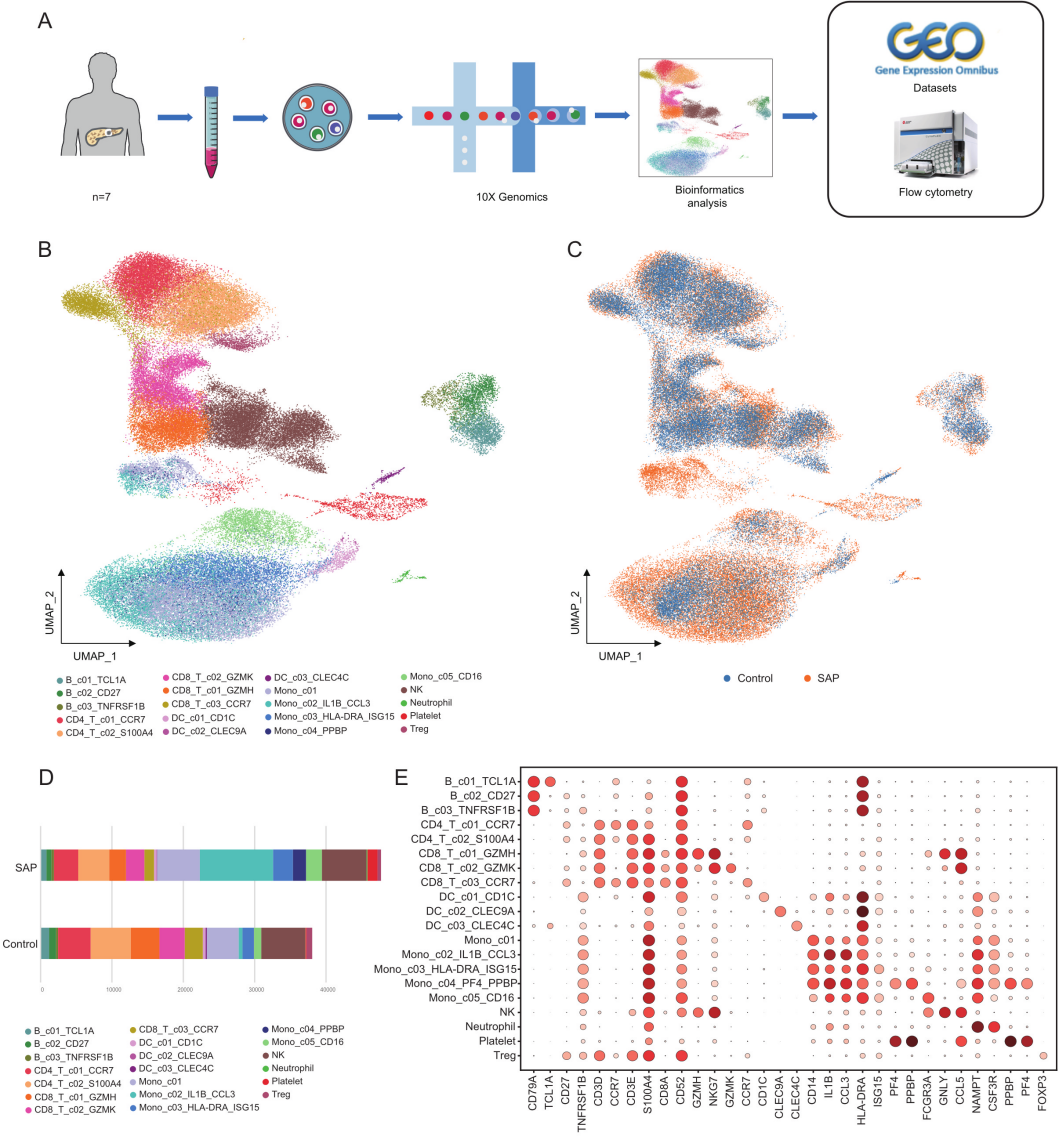
ScRNA-seq transcriptome analysis of cell clusters in SAP patients and healthy controls. **(A)** The study’s workflow diagram. **(B)** Uniform manifold approximation and projection (UMAP) embedding of integrated single-cell transcriptomes of SAP and healthy control samples. Cells are colored by subtype. **(C)** Visualization of SAP dataset vs. healthy control dataset using UMAP embedded graphs. **(D)** Comparison of the percentages of monocyte clusters between SAP patients and controls. **(E)** Dot plots of canonical markers (columns) for major cell types (rows). Control, healthy controls; SAP, severe acute pancreatitis.

To profile the cell types and single-cell gene expression patterns in PBMCs from SAP patients, PBMCs were isolated from 7 SAP patients and subjected to single-cell RNA sequencing technology (10x Genomics). Additionally, single-cell transcriptomic data from 6 healthy donors were obtained from the GEO database and integrated with the SAP patient data for further biological analysis. Flow cytometry validation was also conducted (**Figure 1A**). This study identified a total of twenty cell clusters (**Figure 1B**). When comparing and visualizing the data from healthy controls with those from SAP patients, a significant increase in the number of circulating immune cells was observed in SAP patients (**Figure 1C**), primarily concentrated in monocytes and platelets. In contrast, T cells were notably reduced in SAP patients (**Figure 1D**), the cell numbers and proportions for each subpopulation in healthy controls and SAP patients are presented in **Supplementary Table S4**. The marker genes for each cell cluster are displayed (**Figure 1E**). Existing research has indicated that SAP patients exhibit innate immune cell deficiencies, characterized by a significant decrease in T lymphocytes and a noteworthy increase in monocyte numbers (26, 27). Our findings align with this pattern and suggest that T cell depletion and monocyte expansion may play a pivotal role in SAP. Of particular note, this study for the first time identified a CD14+/CD16- monocyte subset with high expression of PF4 and PPBP, genes typically highly expressed in platelets (28, 29). The discovery of these genes in monocytes is surprising. PF4 and PPBP can mediate systemic inflammatory responses and are potential therapeutic targets for various inflammatory diseases (30, 31). Investigating whether the CD14+/CD16- monocyte subset characterized by high PF4 and PPBP expression also mediates inflammation in SAP patients and its biological significance has become a key research focus for us.

### 3.2 The T cell subsets have significantly decreased in SAP

**Figure 2:**
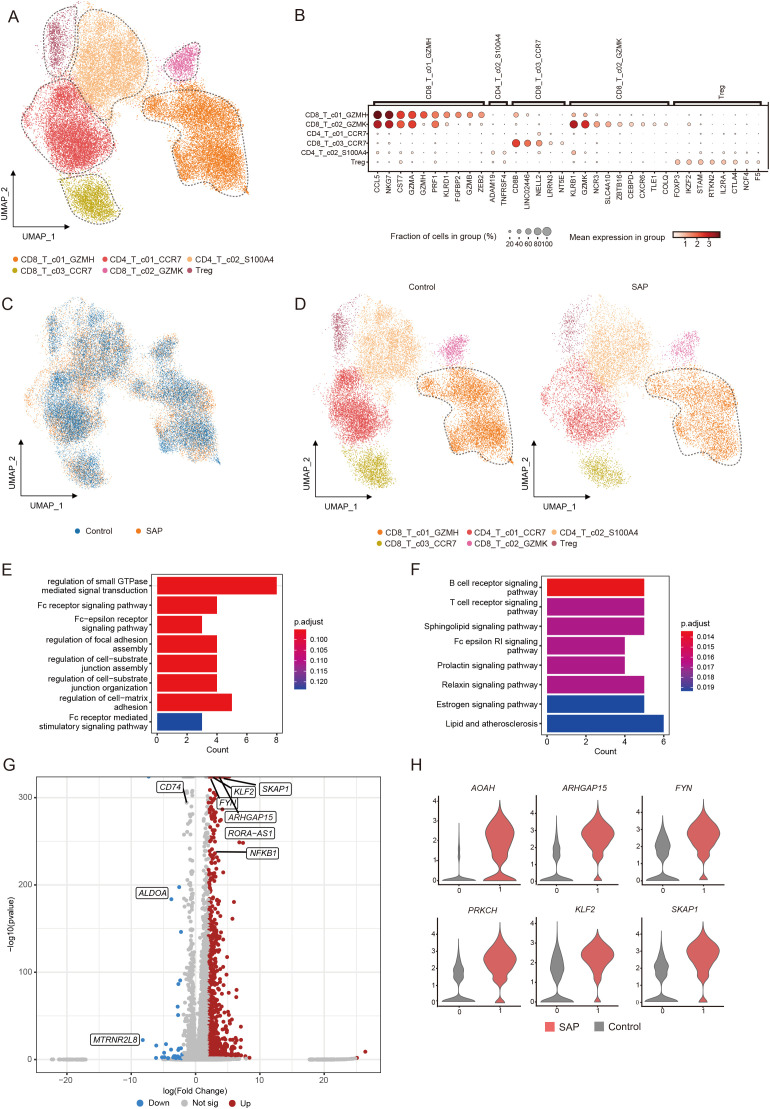
Identification of transcriptomic changes in specific T cell subsets of SAP by transcriptome analysis of scRNA-seq. **(A)** UMAP visualization of transcriptional heterogeneity in human T cells. T cells are further categorized into six subsets, with their respective names provided at the bottom of the graph. Different colors are employed to differentiate between each cluster, with dashed circles indicating the primary cell types. **(B)** Dot plot demonstrates the expression percentage and expression levels of DEGs across various T cell clusters. **(C)** Comparison of SAP dataset and healthy control dataset using UMAP embedded graphs. **(D)** Visualization of T cell clusters in SAP patients and healthy controls using the UMAP downscaling approach. **(E, F)** Main enriched GO and KEGG pathways for significant DEGs in the CD8^+^T_c01_GZMH cluster using cluster Profiler. **(G)** Volcano plots illustrate the DEGs identified in the CD8^+^T_c01_GZMH cluster, with upregulated genes marked in red and downregulated genes in blue. **(H)** Violin plot depicts the expression patterns of DEGs within the CD8^+^T_c01_GZMH cluster in SAP patients and healthy controls. Control, healthy controls; SAP, severe acute pancreatitis.

We further divided T cells into six subpopulations to investigate alterations in T cell subpopulations in SAP (**Figure 2A**). The marker genes for each T cell clusters are presented in Figure. (**Figure 2B**.) In comparison to the healthy control group, all T cell subpopulations displayed significant reductions. Among them, the most noteworthy reduction was observed in the CD8^+^ T cell subset with heightened *GZMH* expression, particularly in the effector CD8^+^ T cell population (**Figures 2C, D**). To comprehend the functions of differentially expressed genes within the considerably diminished effector CD8^+^ T cell population, we conducted comprehensive analyses, encompassing GO pathway enrichment and KEGG pathway enrichment assessments. The outcomes unveiled enrichments in pathways related to small GTPase-mediated signaling regulation, Fc receptor signaling pathways, and B-cell receptor-related pathways in SAP patients (**Figures 2E, F**). This suggests the pivotal role played by these immune and inflammation-related pathways in the pathogenesis of SAP. Further examination of this subpopulation revealed significant upregulation of genes such as *SKAP1*, *KLF2*, *FYN*, *PRKCH*, and others in the CD8^+^ effector T cells of SAP patients (**Figures 2G, H**). These findings indicate substantial alterations in gene expression induced by SAP.

### 3.3 The monocyte subsets with high expression of *PPBP* or *CCL3* are significantly expanded in SAP

**Figure 3:**
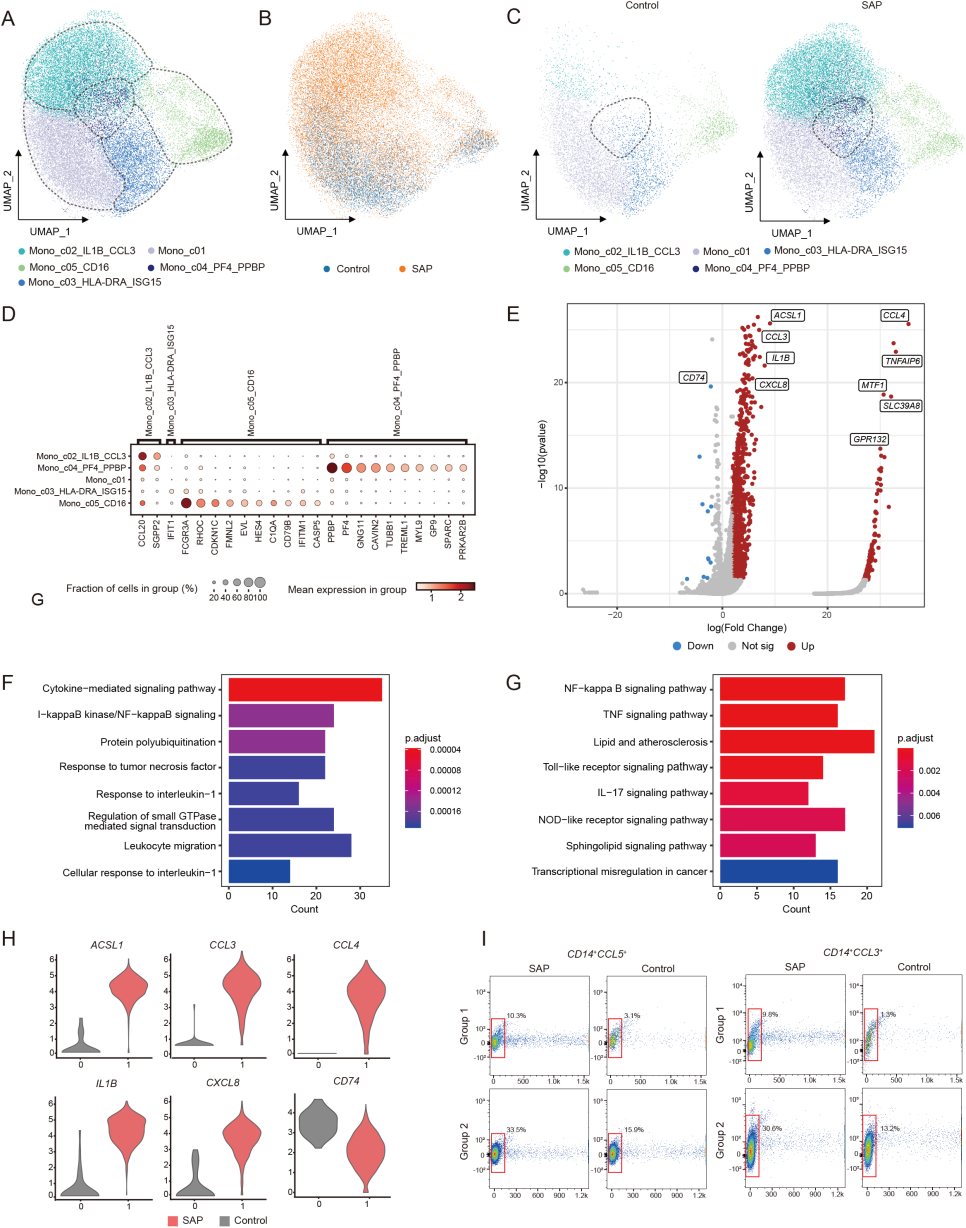
Identification of transcriptomic changes in specific monocyte subsets of SAP by transcriptome analysis of scRNA-seq. **(A)** UMAP visualization of transcriptional heterogeneity in human monocytes. Monocytes are further categorized into five subsets, with their respective names provided at the bottom of the graph. Different colors are employed to differentiate between each cluster, with dashed circles indicating the primary cell types. **(B)** Comparison of SAP dataset and healthy control dataset using UMAP embedded graphs. **(C)** Visualization of monocyte clusters in SAP patients and healthy controls using the UMAP downscaling approach. **(D)** Dot plot demonstrates the expression percentage and expression levels of DEGs across various monocyte clusters. **(E)** Volcano plots illustrate the DEGs identified in the Mono_c04_PF4_PPBP cluster, with upregulated genes marked in red and downregulated genes in blue. **(F, G)** Main enriched GO and KEGG pathways for significant DEGs in the Mono_c04_PF4_PPBP cluster using cluster Profiler. **(H)** Violin plot depicts the expression patterns of DEGs within the Mono_c04_PF4_PPBP cluster in SAP patients and healthy controls. **(I)** Flow cytometry validation was performed by stacked plots for the CCL3^+^/CD14^+^ monocyte subset and the CCL5^+^/CD14^+^ monocyte subset in both SAP and healthy control sample. Control: healthy controls, SAP: severe acute pancreatitis.

Similarly, we further divided the monocytes into five subpopulations and identified them based on high variable genes to investigate changes in monocyte subpopulations in SAP (**Figure 3A**). In comparison to the healthy control group, all monocyte subpopulations displayed significant expansion. Particularly, the subpopulation characterized by high expression of *PF4* and *PPBP* was uniquely expressed in SAP (**Figures 3B, C**). This implies the potential of this subpopulation as a biological marker for SAP and emphasizes its pivotal role in the onset of SAP. The marker genes for each monocyte cluster are presented in the figure (**Figure 3D**). To delve into the functions of DEGs within the significantly expanded monocyte subpopulation exhibiting high expression of *PF4* and *PPBP*, we conducted GO and KEGG pathway enrichment analyses (**Figures 3F, G**). These analyses unveiled enrichments in cytokine-mediated signaling pathways, NF-κB signaling pathways, TNF signaling pathways, Toll-like receptor signaling, and other inflammatory signaling pathways among SAP patients. This further underscores the substantial impact of activated inflammatory signaling pathways on SAP development. Further examination of this subpopulation revealed a significant upregulation of pro-inflammatory cytokines in SAP patients, such as *CCL3*, *IL1B*, and *CXCL3* (**Figures 3E, H**). Previous research has confirmed the biological significance of these cytokines in predicting the occurrence and progression of SAP, a conclusion supported by our study. To validate the significant expansion of the two cell clusters, we utilized flow cytometry. Due to the difficulty in obtaining antibodies for PPBP and PF4 suitable for flow cytometry experiments, we selected CCL5, which is also highly expressed in this subset and lowly expressed in other subsets, as a marker for this population. CCL3 was used to identify the mono_c02_IL1B_CCL3 subset. Our results indicated a significant increase in the proportion of the monocyte subpopulation with high expression of *PF4* and *PPBP*, as well as the monocyte subpopulation with high expression of *IL1B* and *CCL3*, in monocytes of SAP patients when compared to healthy controls (**Figure 3I**). This aligns with our findings from the single-cell transcriptome analysis. In summary, the monocyte findings are consistent with prior research, emphasizing their crucial role in the progression of SAP.

### 3.4 Cell communication reveals the pivotal role of the immune system in SAP

**Figure 4:**
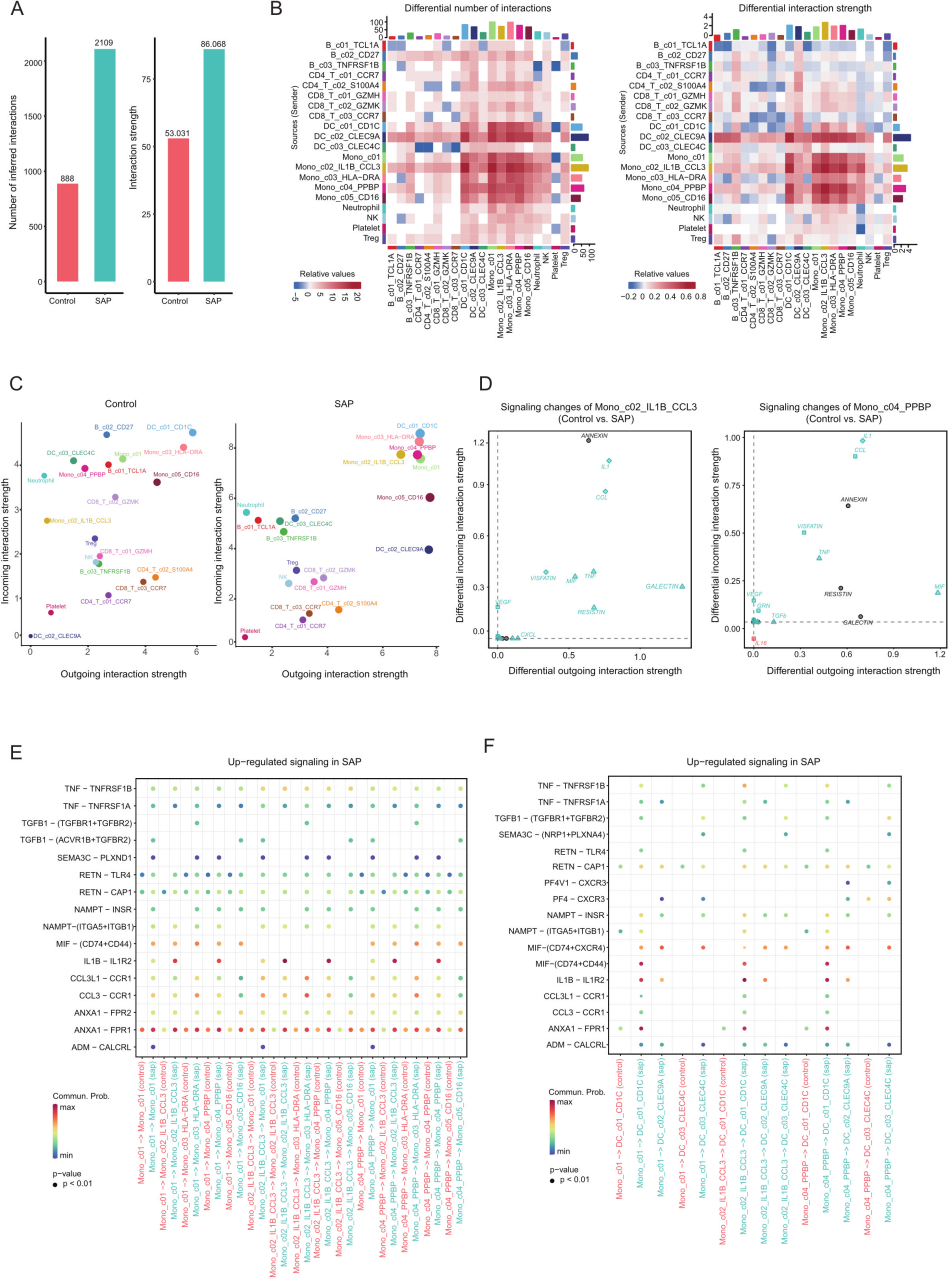
Inference and analysis of cell-cell communication using CellChat. **(A)** The bar chart illustrates both the total (left) and interaction strength (right) of ligand-receptor interactions between distinct cell subpopulations in SAP patients and the control group. **(B)** The heatmap offers a more comprehensive representation of the quantity and intensity of interactions among diverse subpopulations. The colored bars at the top present the cumulative values for each column as indicated in the heatmap (incoming signals). The colored bars on the right depict the sum of values for each row (outgoing signals). In the color bars, red (or blue) signifies an augmentation (or reduction) in signals within the SAP dataset compared to the control dataset. **(C)** A comparative analysis of the intensity of inbound and outbound interactions within individual subpopulations between the SAP dataset and the control dataset is presented. The dot size reflects the volume of communication. **(D)** Evaluation of alterations in cell communication within two monocyte subpopulations featuring significantly elevated interaction strengths in the SAP dataset in contrast to the control dataset. **(E, F)** An assessment of considerably enhanced signals among diverse monocyte subpopulations and between monocyte subpopulations and DC cell subpopulations in the SAP dataset and the control dataset. Empty cells denote a zero probability of communication. Control: healthy controls, SAP: severe acute pancreatitis.

To gain deeper insights into the disparities in immune cell-cell interactions between patients with SAP and healthy controls, we employed the Cell Chat package for an extensive analysis of major signaling inputs and outputs from each cell cluster (24), along with the pathways through which signaling sources and signaling pathways synergize in their functions. Between the various cell types observed in SAP patients and healthy controls, we identified a total of 2109 and 888 significant ligand-receptor (LR) interactions, respectively. Notably, our analysis unveiled significantly higher interaction numbers and strengths within the SAP dataset when compared to the healthy control dataset (**Figure 4A**). Noteworthy is the dominant contribution of the monocyte cluster to these differences, affirming our previous research findings that underscore the pivotal role of the monocyte cluster in the onset of SAP (**Figure 4B**). Furthermore, our analysis unveiled LR interactions with the most pronounced input and output signals occurring in monocytes expressing elevated levels of *IL1B* and *CCL3*, as well as monocytes with heightened *PF4* and *PPBP* expression (**Figure 4C**). Additionally, signaling factors related to inflammation such as *IL1*, *TNF*, *MIF*, *CCL*, and *VISFATIN* were uniquely observed in the SAP dataset, participating in both input and output signaling processes (**Figure 4D**). Previous studies have demonstrated that cytokines like *IL1*, *TNF*, and *MIF* can serve as predictors of the severity of AP (32), suggesting the potential utility of these genes as biological markers for SAP. This implies that these genes hold promise as potential biological markers for SAP. Moreover, when examining receptor-ligand interactions across different cell clusters in the two single-cell datasets, we observed substantial differences in the patterns between the two datasets. Specifically, immune cells in SAP patients displayed a greater number of altered immune pathways, with a noticeable upregulation of inflammation-related ligand-receptor pairs, including MIF-(CD74+CD44), IL1B-IL1R2, and CCL3-CCR1 within the SAP group (both among different monocyte clusters and between different monocyte clusters and dendritic cell clusters) (**Figures 4E, F**). In conclusion, these identified genes and pathways underscore the critical roles played by both the innate and adaptive immune systems in SAP.

### 3.5 The monocyte subset with high *CCL3* expression is significantly associated with disease severity

**Figure 5:**
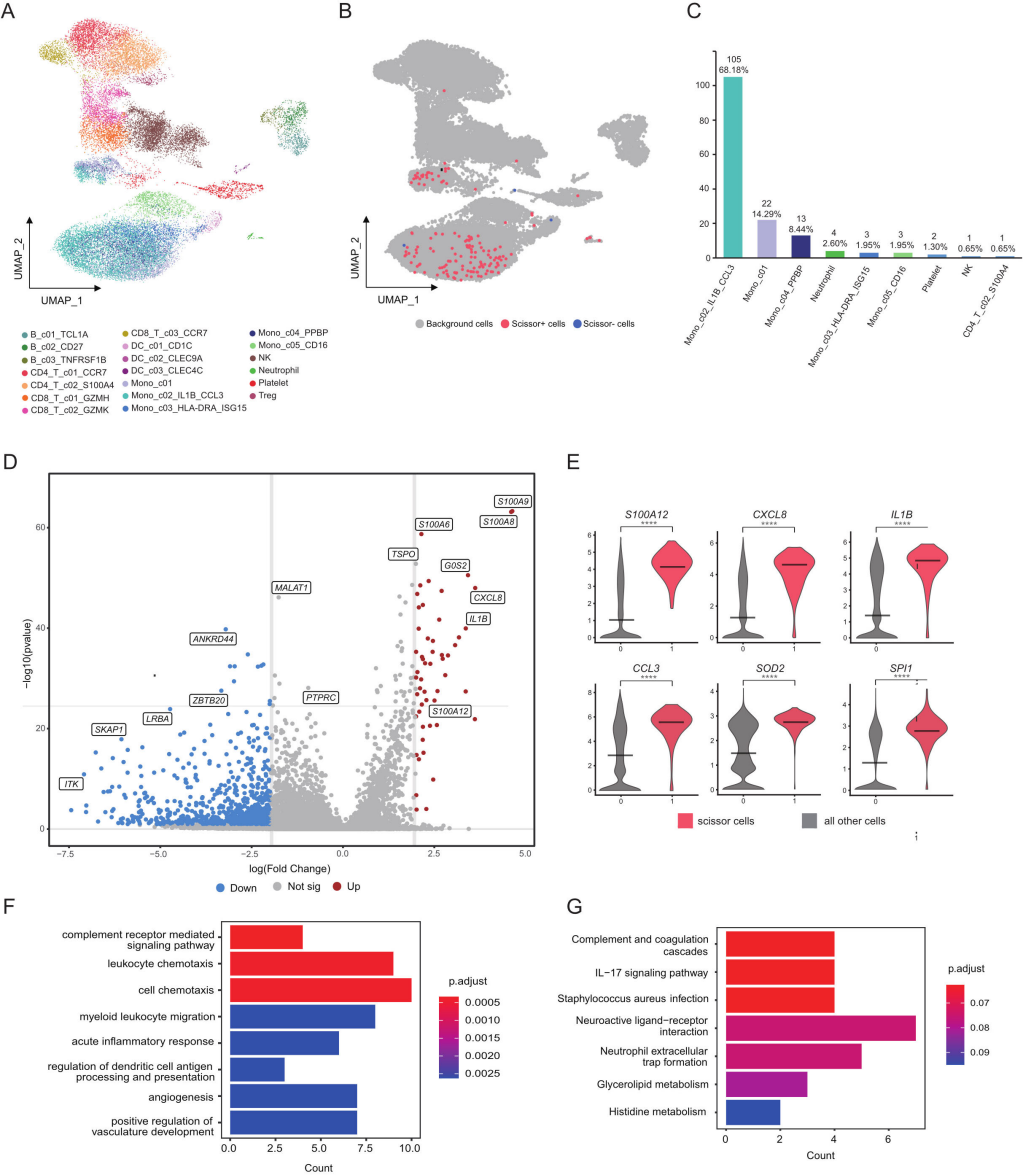
Cellular subpopulation profiles with disease severity in acute pancreatitis. **(A)** Visualization of cellular clusters within the SAP dataset using UMAP dimensionality reduction. **(B)** Scissor cells identified in the SAP dataset through UMAP dimensionality reduction. Red and blue points correspond to scissor+ and scissor- cells, respectively. **(C)** Bar chart depicting the quantity and proportion of scissor+ cells across various subgroups. **(D)** Volcano plots illustrate the DEGs identified in the Mono_c02_IL1B_CCL3 cluster, with upregulated genes marked in red and downregulated genes in blue. **(E)** Violin plot depicts the expression patterns of DEGs within the Mono_c02_IL1B_CCL3 cluster in SAP patients and healthy controls. **(F, G)** Main enriched GO and KEGG pathways for significant DEGs in the Mono_c02_IL1B_CCL3 cluster using cluster Profiler. Control: healthy controls, SAP: severe acute pancreatitis.

To further enhance our understanding of the distinctions in immune cell interactions between SAP patients and healthy controls, we integrated the single-cell dataset with the bulk transcriptome dataset using the “Scissor” tool, facilitating a comprehensive analysis of the previously mentioned 20 distinct cell clusters (**Figure 5A**). In the course of this investigation, we pinpointed a total of 157 Scissor cells that exhibited associations with disease severity. Among these, 154 Scissor cells were linked to more severe disease conditions, categorized as Scissor^+^ cells, while a mere 3 cells were associated with milder disease states, termed Scissor^-^ cells (**Figure 5B**). We performed rigorous reliability tests of the Scissor commands, with results confirming their trustworthiness (*p* < 0.05). Significantly, Scissor^+^ cells are predominantly concentrated within the subgroup of monocytes displaying elevated levels of *IL1B* and *CCL3* expression (**Figure 5C**). This finding strongly suggests the significant role played by monocytes in disease severity. Within this subgroup, we identified 54 upregulated genes when compared to background cells, which are cells not associated with disease severity. Notable genes in this context included *S100A12*, *S100A9*, *CXCL3*, and *IL-1B* (**Figures 5D, E**). These observations emphasize the central role of immune-related genes in predicting the severity of Acute Pancreatitis. As supported by previous studies, genes like *S100A12*, *S100A9* and *IL-1B* hold substantial value as markers for predicting the severity of Acute Pancreatitis, in line with our research results (33). Subsequently, we conducted a functional analysis of the differentially expressed genes. These genes were notably enriched in pathways related to inflammation and immunity, including complement-related pathways, chemotaxis-related pathways and inflammation-related pathways (**Figures 5F, G**). These findings underscore the crucial role played by these pathways in the progression of Acute Pancreatitis to a severe stage.

## 4 Discussion

This study marks the first comprehensive analysis of immunocyte transcriptome data from the peripheral blood of 7 SAP patients employing scRNA-Seq technology. Our findings underscore a notable depletion of both T cells and B cells within SAP patients, alongside a significant expansion of monocyte subpopulations. These observations parallel earlier studies, notably those by Dabrowski and others employing flow cytometry, collectively underlining a pronounced disruption of innate immune cell populations in SAP patients (27). Research suggests that dysfunction of lymphocytes plays a significant role in the complications associated with SAP infection (34), The increased presence of monocytes plays a pivotal role in mediating the occurrence of SAP (35).

Our research outcomes propose that the depletion of T cells, a factor that exacerbates SAP progression, may be linked to the substantial upregulation and activation of genes and related pathways that curtail T cell functionality. Earlier research indicates the therapeutic potential of inhibiting *AOAH* (36). The notably activated small GTPase activation-related pathways play a critical role in mediating the diminished activity of T cells (37). Activation of Fc receptor-related pathways indirectly heightens the production of neutrophil extracellular traps (NETs) in neutrophils, intensifying local damage to pancreatic tissue and contributing to systemic inflammatory response syndrome (38–40). Increased platelets are also recruited and encourage NETs formation, collectively participating in this destructive process (41–43). To summarize, these findings highlight a positive correlation between the depletion of T cells and SAP progression, offering fresh insights into halting SAP advancement and introducing new prospects for enhancing immune function in SAP treatment.

This study has, for the first time, identified a unique subpopulation of CD14^+^/CD16^-^ monocytes characterized by high expression of *PF4* or *PPBP*. This subpopulation not only exhibits a significant differential gene expression but also possesses distinct specific characteristics. *PF4* can mediate systemic inflammatory responses (30). while *PPBP* is closely associated with inflammation, positioning it as a potential biomarker for elevated inflammation in a range of diseases (31, 44). In addition, reports also suggest that *PPBP* is specifically expressed in various infectious diseases (45, 46). And our future research will delve further into its pivotal role in infectious diseases by utilizing large sample datasets. Within this subpopulation, we observed a significant upregulation and activation of genes and pathways related to inflammation, with *IL1B* showing a positive correlation with the severity of acute pancreatitis (47). *CCL3* induces inflammation in acute pancreatitis by upregulating chemokine receptor 1/chemokine receptor 5 (48). Activated cytokine signaling pathways (49), NF-κB signaling pathways (50) and TNF signaling pathways (51) can incite and expedite systemic inflammatory response syndrome. Significantly, experimental studies have indicated the potential advantages of targeting TNF (49, 51), reducing leukocyte infiltration, and curtailing cytokine release (52) in experimental therapies for pancreatitis. This study also uncovered an increased subpopulation of monocytes with high expression of *HLA-DRA* and *ISG15*, the latter being a specific transcriptional product of the pro-inflammatory monocyte subpopulation significantly associated with treatment efficacy (53).

Using the “CellChat” package for cell-cell communication analysis, we unveiled that interactions between cells in SAP patients, both in terms of quantity and intensity, were significantly more pronounced than in healthy controls. This phenomenon was most conspicuous in interactions among monocytes themselves and in interactions between monocytes and dendritic cells. Notably, ligand-receptor pairs such as TNF-TNFRSF1B and IL1B-IL1R2 exhibited heightened activity in monocyte interactions with other cells, aligning with the activated pathways and heightened inflammatory status within SAP patient monocytes. Furthermore, we identified that the ligand macrophage migration inhibitory factor (MIF) and its CD74 receptor were highly active, leading to the increased production of pro-inflammatory cytokines such as IL-6, IL-8, TNFα, and IL-1β. This, in turn, generated an inflammatory microenvironment, exacerbating the progression of the inflammatory storm (54), and is an important pathway for SAP to develop (15). Previous reports have suggested that reducing the interaction of MIF-CD74 could significantly alleviate the inflammatory effects induced by pro-inflammatory factors (55).

Finally, we conducted an analysis of subpopulations of cells significantly associated with disease severity and uncovered crucial expressions of inflammation-related genes in the progression of AP. Among these, *S100A8* and *S100A9* were found to accurately predict SAP severity with high precision (15). Additionally, *IL1B* (56) and *CCL3* (48) have been reported as promising biomarkers for predicting SAP. Furthermore, pathways linked to complement receptor-mediated signaling, leukocyte chemotaxis, and cell migration were activated in Scissor^+^ cells. Reports indicate that complement receptor-mediated signaling can promote organ damage in SAP, highlighting the therapeutic value of complement inhibition (57). Furthermore, pathways linked to complement receptor-mediated signaling, leukocyte chemotaxis, and cell migration were activated in Scissor^+^ cells. Reports indicate that complement receptor-mediated signaling can promote organ damage in SAP, highlighting the therapeutic value of complement inhibition (58). As such, the increased platelet count in SAP patients not only interacts with leukocytes but also collectively contributes to SAP progression. These findings underscore the complexity and significance of the immune environment in SAP, where various cell subpopulations have a cascading amplification of the inflammatory effect, collectively intensifying systemic inflammation in SAP.

In summary, this study, through the utilization of scRNA-seq for SAP analysis, has once again affirmed the pivotal role of immune cells in SAP. Moreover, it has unveiled potential therapeutic targets within the upregulated genes and activated pathways, providing novel insights into potential SAP treatment strategies and introducing a new indicator for predicting the occurrence and progression of SAP. However this study involved a limited number of SAP samples, and samples were not categorized based on the etiology of SAP. In our future work, we plan to expand the sample size and investigate the various etiologies contributing to SAP.

## Data Availability

The original codes used for analyses presented in the study are publicly available. This data can be found here: https://github.com/Docwuzheyi/WuZheyi. The datasets presented in this study can be found in online repositories. The names of the repository/repositories and accession number(s) can be found below: HRA006290 (GSA; https://ngdc.cncb.ac.cn/search/?dbId=hra&q=HRA006290).
