# Supplementary material for: Altered immune cell in human severe acute pancreatitis revealed by single-cell RNA sequencing
Source: Front Immunol. 2024 Sep 20;15:1354926. doi: 10.3389/fimmu.2024.1354926 (PMC11449708; doi:10.3389/fimmu.2024.1354926)
Supplement: Supplementary file 1 [file Table1.docx]

# Supplementary Table S1 General characteristics of healthy controls and severe acute pancreatitis patients, related to Figure 1

| Characteristics | scRNA-seq | | flow cytometry | |
| --- | --- | --- | --- | --- |
|  | Control | SAP | Control | SAP |
| Number of cases | 6 | 7 | 2 | 2 |
| Age (year range) | 62(51-74) | 58(52-65) | 47(46-48) | 47(44-50) |
| Gender (male/female) | 2/4 | 2/5 | 1/1 | 1/1 |
| WBC (10^9^/L) | NA | 15.7(11.7-22.6) | 4.4(4.1-4.7) | 1.36(10.7-16.5) |
| Neutrophils (10^9^/L) | NA | 13.9(10.6-19.6) | 2.4(2.3-2.5) | 12.7(10.1-15.3) |
| Lymphocytes (10^9^/L) | NA | 0.8(0.5-1.1) | 1.6(1.3-1.9) | 0.6(0.5-0.7) |
| Monocytes (10^9^/L) | NA | 0.7(0.4-1.3) | 0.35(0.3-0.4) | 0.5(0.3-0.7) |
| AMY (U/L) | NA | 782(328-1116) | 41(37-45) | 924(848-1000) |
| Ca (mmol/L) | NA | 2.24 (1.87-2.43) | 2.29(2.27-2.31) | 1.43(2.35-2.51) |
| CRP (g/L) | NA | 146(57-221) | 0.5(0.4-0.6) | 109 (87-131) |
| APACHE II score | NA | 14(11-17) | NA | 10(7-13) |
| LOS (d) | NA | 12(8-17) | NA | 8(7-11) |

Data shown are median (range) or number of cases. Control, healthy controls; SAP, severe acute pancreatitis patients; WBC, white blood cell count; AMY, amylase; CRP, C-reactive protein; APACHE, acute physiology and chronic health evaluation; NA, not available; LOS, length of hospital stay. normal values: WBC: 4.0–10.0 (10^9^/L), neutrophils: 2.0–7.0(10^9^/L), lymphocytes: 0.8–4.0 (10^9^/L), monocytes: 0.12–0.80 (10^9^/L), AMY: 8–220 (U/L), Ca 2.25–2.67(mmol/L), and CRP: 0–10 (mg/L).
