# Supplementary material for: Altered immune cell in human severe acute pancreatitis revealed by single-cell RNA sequencing
Source: Front Immunol. 2024 Sep 20;15:1354926. doi: 10.3389/fimmu.2024.1354926 (PMC11449708; doi:10.3389/fimmu.2024.1354926)
Supplement: Supplementary file 4 [file Table4.docx]

Supplementary Table S4 Cell populations and ratio of each cell clusters in healthy controls and severe acute pancreatitis patients, related to Figure 1

| Cell clusters | Control | | SAP | |
| --- | --- | --- | --- | --- |
|  | Cell populations | Ratio | Cell populations | Ratio |
| B_c01_TCL1A | 1205 | 3.17% | 821 | 1.72% |
| B_c02_CD27 | 965 | 2.54% | 725 | 1.52% |
| B_c03_TNFRSF1B | 279 | 0.73% | 323 | 0.68% |
| CD4_T_c01_CCR7 | 4566 | 12.00% | 3403 | 7.13% |
| CD4_T_c02_S100A4 | 5627 | 14.78% | 4354 | 9.13% |
| CD8_T_c01_GZMH | 4018 | 10.56% | 2342 | 4.91% |
| CD8_T_c02_GZMK | 3554 | 9.34% | 2576 | 5.40% |
| CD8_T_c03_CCR7 | 2532 | 6.65% | 1359 | 2.85% |
| DC_c01_CD1C | 370 | 0.97% | 338 | 0.71% |
| DC_c02_CLEC9A | 20 | 0.05% | 38 | 0.08% |
| DC_c03_CLEC4C | 171 | 0.45% | 56 | 0.12% |
| Mono_c01 | 4464 | 11.73% | 5989 | 12.55% |
| Mono_c02_IL1B_CCL3 | 523 | 1.37% | 10271 | 21.53% |
| Mono_c03_HLA-DRA_ISG15 | 1591 | 4.18% | 2770 | 5.81% |
| Mono_c04_PPBP | 40 | 0.11% | 1844 | 3.86% |
| Mono_c05_CD16 | 1002 | 2.63% | 2217 | 4.65% |
| NK | 6164 | 16.19% | 6261 | 13.12% |
| Neutrophil | 29 | 0.08% | 128 | 0.27% |
| Platelet | 153 | 0.40% | 1399 | 2.93% |
| Treg | 791 | 2.08% | 497 | 1.04% |

Control, healthy controls; SAP, severe acute pancreatitis.
